# HPV and co-infections: impacts on semen inflammation, oxidative stress, and sperm quality

**DOI:** 10.3389/fcimb.2025.1539871

**Published:** 2025-03-26

**Authors:** Carolina Olivera, Daniela A. Paira, Andres Olmedo, Jose J. Olmedo, Andrea D. Tissera, Rosa I. Molina, Fernando N. Ferreyra, Maria S. Martinez, Yair A. Chocobar, Cecilia G. Cuffini, Ruben D. Motrich, Virginia E. Rivero

**Affiliations:** ^1^ Federation of Clinical Immunology Societies (FOCIS) Center of Excellence, Centro de Inmunología Clínica de Córdoba (CICC), Córdoba, Argentina; ^2^ Centro de Investigaciones en Bioquimica Clinica e Inmunologia-Consejo Nacional de Investigaciones Cientificas y Tecnicas (CIBICI-CONICET), Facultad de Ciencias Químicas, Universidad Nacional de Córdoba, Córdoba, Argentina; ^3^ Dirección de Asistencia Social del Personal Universitario (DASPU), Universidad Nacional de Córdoba, Córdoba, Argentina; ^4^ Fundación Urológica Córdoba para la Docencia e Investigación Médica (FUCDIM), Córdoba, Argentina; ^5^ Laboratorio de Andrología y Reproducción (LAR), Córdoba, Argentina; ^6^ Instituto de Virología Dr. José M. Vanella, Facultad de Ciencias Médicas, Universidad Nacional de Córdoba, Córdoba, Argentina

**Keywords:** HPV, *Chlamydia trachomatis*, infection, semen, inflammation, fertility

## Abstract

**Introduction:**

Human papillomavirus (HPV) is the most common sexually transmitted viral infection worldwide, which has been suggested to induce male urogenital inflammation and affect fertility. However, reported evidence is scarce and inconclusive. Moreover, the putative effects of coinfections remain largely unexplored. This study aimed to analyze HPV male urogenital infection, both as a single infection and in conjunction with other common uropathogens, along with its impact on inflammatory biomarkers in semen, oxidative stress and sperm quality.

**Methods:**

The prevalence of HPV urogenital infection and its coinfection with several other uropathogens was analyzed in a cohort of 205 men attending a urology clinic. Furthermore, levels of sperm leukocyte subsets and inflammatory cytokines, semen analysis, sperm apoptosis and necrosis, and sperm ROS production were assessed.

**Results:**

A considerable prevalence (19%) of HPV male urogenital infection was found. Interestingly, HPV was detected coinfecting with at least one other uropathogen in most cases (74.4%). Notably, the most frequently detected coinfection was *C. trachomatis* (CT, 52% of cases). Remarkably, patients solely infected with HPV showed no significant alterations in conventional sperm quality parameters as well as reduced concentrations of IL-6 and leukocytes in semen. However, patients coinfected with HPV and CT showed significantly impaired sperm concentration and motility and increased levels of IL-6 in semen.

**Conclusion:**

These results indicate that HPV infection alone does not associate with semen inflammation or major changes in sperm quality. However, co-infection with CT is associated with both semen inflammation and reduced sperm quality. This indicates that, besides being prevalent, concurrent HPV and CT infections in semen may represent a unique clinical entity with particular characteristics.

## 1 Introduction

Sexually transmitted infections (STIs) are a major public health problem worldwide. Genitourinary pathogens are frequently detected either as single infection or coinfections reflecting the complexity of their transmission dynamics (Dalby and Stoner, 2022; Lee et al., 2023). Although these infections frequently go undetected and persist unnoticed, they can lead to long-term sequelae and increased morbidity (Dalby and Stoner, 2022).

Human papillomavirus (HPV) is the most common sexually transmitted viral infection in males and females worldwide. HPV infection represents a significant public health concern globally, with its implications extending beyond cervical cancer to encompass a spectrum of diseases affecting both sexes (Doorbar et al., 2015). In women, several studies have reported that the concurrent detection of other uropathogens such as *Chlamydia trachomatis* (CT), *Trichomonas vaginalis* (TV), or *Ureaplasma* spp. seems to increase the likelihood of HPV persistence and may contribute to the female reproductive system pathology (Alotaibi et al., 2020; Cunha et al., 2020; Ciccarese et al., 2021). Among these, CT is considered the main cofactor associated with the progression of cervical cancer in HPV infected women (de Abreu et al., 2016; Naldini et al., 2019; Alotaibi et al., 2020; Chen et al., 2020). In men, HPV infection often manifests as anogenital warts and in some cases is associated with penile, anal, and oropharyngeal cancers (Moscicki, 2011; Sarier et al., 2020). However, the impact of HPV infection on male reproductive health and its relationship with other STIs remains controversial (Gimenes et al., 2014a; Pérez-González et al., 2022). While growing evidence suggests that HPV may have diverse effects on the male reproductive system, reported data about consequences of HPV infection in semen is controversial (Moreno-Sepulveda and Rajmil, 2021). Indeed, some studies have associated the infection with reduced sperm quality, mainly decreasing sperm concentration and sperm motility, and causing adverse reproductive outcomes (Boeri et al., 2019; Depuydt et al., 2019; Cao et al., 2020). However, others studies reported contradicting findings (Rintala et al., 2004; Schillaci et al., 2013; Golob et al., 2014). Moreover, there is a lack of comprehensive data on whether HPV male urogenital infection associates with local inflammation (Chihu-amparan et al., 2023). In that regard, leukocytospermia has been widely used in fertility clinic as a biomarker of inflammation/infection (Henkel et al., 2021). Nevertheless, increased levels of reactive oxygen species (ROS) and inflammatory cytokines have been recognized as reliable indicators of genital tract oxidative stress and inflammation able to impair male fertility (Agarwal et al., 2018; Henkel et al., 2021).

In this context, coinfection scenarios must be taken into account, wherein HPV can interact with other uropathogens that may exacerbate pathogenic effects (Ciccarese et al., 2021). Moreover, the simultaneous presence of multiple pathogens in the male reproductive tract may complicate the assignment of the specific role of HPV infection in the observed sperm quality alterations and/or urogenital inflammation (Shigehara et al., 2011; Pérez-Soto et al., 2021a). Differentiating between HPV as a single infection or coinfecting with other uropathogens will help identify specific pathogenic effects attributable to each scenario.

The aim of this study is to characterize the local immune response and inflammatory biomarkers associated with HPV infection of the male urogenital tract, whether it occurs as a single infection or alongside with other common uropathogens, and to examine its effects on semen inflammation, oxidative stress, and sperm quality.

## 2 Materials and methods

### 2.1 Ethics statement

The study was carried out in accordance with the Code of Ethics of the World Medical Association (Declaration of Helsinki) standards and the Argentinian legislation for protection of personal data (Law 25326). The experimental protocol was approved by the Institutional Ethics Committee from Centro Médico Oulton-Romagosa, Córdoba, Argentina (RePIS #3625). All subjects provided a signed written informed consent prior to their enrollment in the study agreeing to share their own anonymous information.

### 2.2 Study design, subjects, and samples

**Figure 1:**
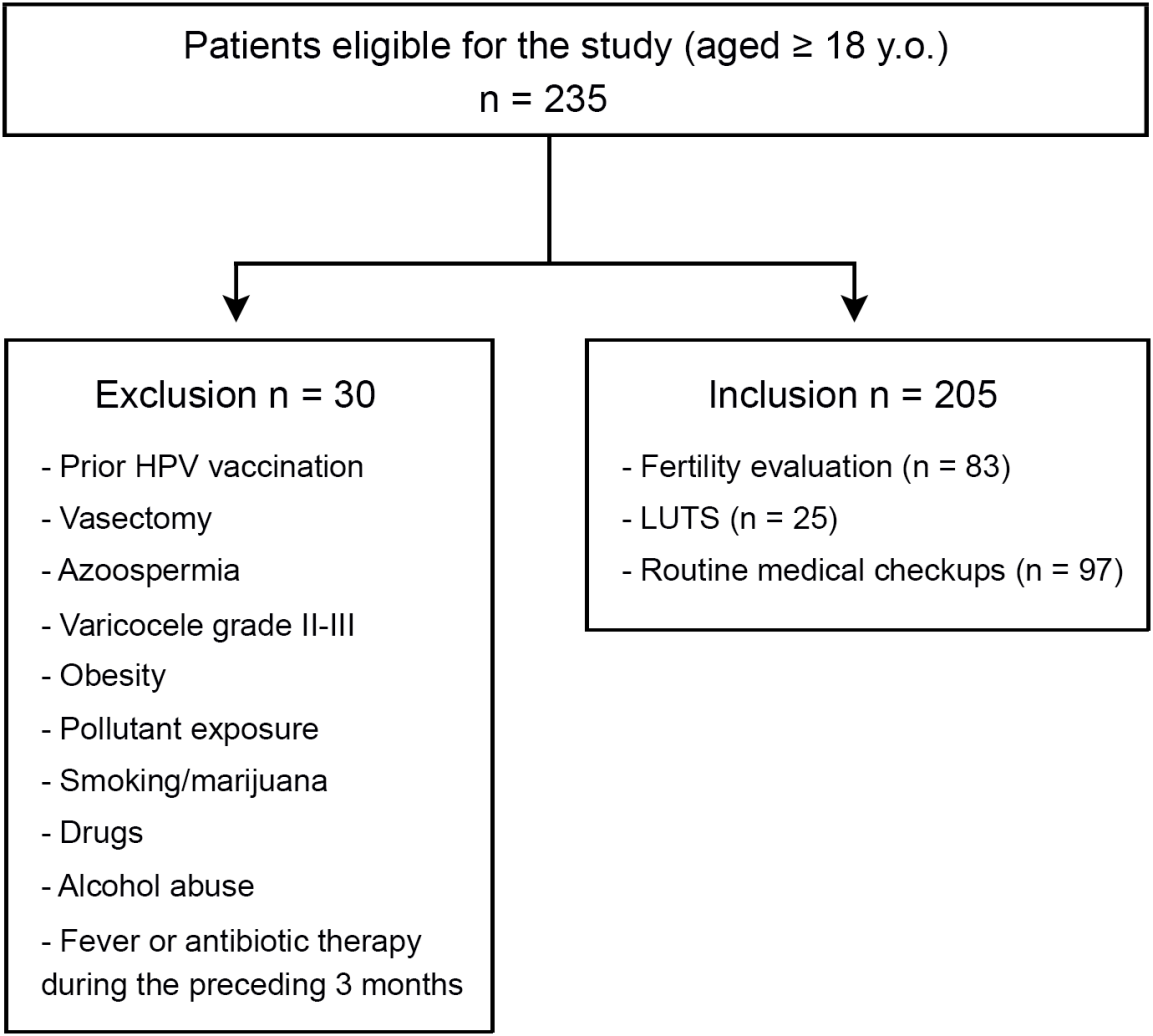
Flowchart of patient enrollment.

This prospective cross-sectional study enrolled a cohort of 235 adult male individuals who attended a combined academic, urology, and andrology clinic from August 2018 to August 2021. Inclusion criteria were: men aged 18 years or older, who underwent a semen analysis as part of their initial fertility evaluation, seeking care for lower urinary tract symptoms (LUTS), or undergoing routine medical checkups. Thirty patients were excluded from the study according to the following criteria: individuals with prior HPV vaccination, vasectomy, azoospermia, varicocele grades 2-3, documented exposure to environmental pollutants (e.g., pesticides), obesity (BMI >30.0), smoking, drug, alcohol, or marijuana use, fever or antibiotic therapy during the preceding 3 months. Thus, 205 patients were included in all the analyses performed (**Figure 1**). All included participants provided semen specimens.

### 2.3 Semen collection

Patients were instructed to thoroughly cleanse their hands and penis before semen sample collection. Semen samples were obtained after 3-5 days of sexual abstinence through masturbation and ejaculation directly into a standard sterile container, which was delivered to the laboratory within 1 h of collection. Samples were liquefied at 37°C for approximately 20 min in an incubator prior to semen analysis. Aliquots of semen specimens were centrifuged at 400 x *g* for 15 min to obtain seminal plasma, which was stored at -80°C until use. The cellular pellet was used to purify total DNA for uropathogens screening by PCR, as described below.

### 2.4 Uropathogens screening

The detection of different common uropathogens in semen was performed by culture or polymerase chain reaction (PCR). Human Papillomavirus, *Chlamydia trachomatis* (CT), *Ureaplasma urealyticum* (UU), *Mycoplasma hominis* (MH), herpes simplex virus (HSV) 1, HSV2, *Mycoplasma genitalium* (MG), *Treponema pallidum* (TP), *Neisseria gonorrhoeae* (NG) and *Trichomonas vaginalis* (TV) were analyzed by PCR as previously described (Paira et al., 2023). Total DNA was extracted from cellular pellets obtained after centrifuging semen aliquots using the AccuPrep Genomic DNA Extraction Kit (BIONEER Corp., Republic of Korea) according to the manufacturer’s instructions. HPV was detected using the consensus primers MY11/MY09, which amplify the L1 region of the viral genome (Olivera et al., 2021). CT was assessed by amplification of the *ompA* gene using the primers SeroA1, SeroA2, pCTM3, and the cryptic plasmid gene using the primers CTP1/CTP2, as previously described (Paira et al., 2023). HSV1, HSV2, MG, TP, NG and TV were assessed by different multiplex PCR adapted from the protocol described by Gimenes et al (Gimenes et al., 2014b). A DNA-free sample was used as negative control and a positive sample for the respective uropathogen was used as a positive control in each case. Co-amplification of the human *β*-globin gene was performed as an internal control using the GH20/PC04 primers under same amplification conditions. The presence of MH and UU was also assessed using the commercially available MYCOFAST Screening RevolutioN colorimetric culture test (cat 00063, ELITech Group) following the manufacturer’s instructions. Additionally, the presence of *Escherichia coli* (EC), *Enterococcus faecalis* (EF), *Proteus mirabilis*, *Enterobacteriaceae*, *Pseudomonas* spp., *Streptococcus* spp.*, Staphylococcus* spp.*, Corynebacteriaceae*, and *Candida* spp. was analyzed by culture in nutrient-rich media (Nutrient agar, MacConkey agar, chocolate agar, and blood agar) incubated for 72 hours at 37°C in a 5% CO_2_ atmosphere. Concurrently, Gram staining and microscopic examination were performed.

### 2.5 Semen analysis

Semen analysis was performed at least twice in each individual according to the World Health Organization Semen Analysis Manual 5^th^ edition (2010) (World Health Organization, 2010). Routine sperm parameters were assessed in at least 200 spermatozoa per sample by two operators, rendering a total of 400 scored spermatozoa.

### 2.6 Sperm apoptosis/necrosis assessment

Sperm apoptosis/necrosis was assessed immediately after semen liquefaction by annexin V (AnV)/propidium iodide (PI) staining and flow cytometry as previously described (Puerta Suarez et al., 2017). **Supplementary Figure S1A** shows the gating strategy used.

### 2.7 Assessment of sperm reactive oxygen species (ROS) production

Sperm ROS production was analyzed as previously described (Puerta Suarez et al., 2017). In brief, intracellular ROS levels were evaluated by flow cytometry using a cell-permeable probe 2’,7’-dichlorodihydrofluorescein diacetate (Dcfh-DA, 1μM; Sigma-Aldrich). This method enables a sensitive quantification of ROS in response to oxidative metabolism. Propidium iodide (PI, Molecular Probes Inc., The Netherlands) was used in conjunction with Dcfh-DA as a supravital stain (final concentration: 12μM). **Supplementary Figure S1B** shows the gating strategy used.

### 2.8 Semen leukocyte analysis

The assessment of leukocyte subsets in semen was performed by flow cytometry as previously described (Paira et al., 2023). Single-cell suspensions were obtained after centrifuging 100 µl of semen specimens for 5 min at 2000 rpm to remove seminal plasma and cells were resuspended in ice cold FACS Buffer (10% FBS supplemented PBS). Cell suspensions were stained with fluorescent labeled antibodies specific to human CD45, CD3, CD4, CD8, and CD20 (BioLegend, San Diego, CA, USA) and CD14, CD11c and CD11b (eBioscience) and analyzed on a FACS Canto II cytometer. The entire sample was acquired in order to calculate the ratio of CD45+ cells per ml of semen. Data were analyzed using the FlowJo 7.6 software (Tree Star, Ashland, OR, USA). As shown in **Supplementary Figure S1C**, the leukocyte (CD45+) subpopulations identified included polymorphonuclear leukocytes (PMN, CD11c- CD11b+), monocytes (CD14+ CD11b-), and lymphocytes (CD11c- CD11b-). T lymphocytes were identified as CD3+ cells, either as CD4 T cells (CD3+ CD4+ CD8-) or CD8 T cells (CD3+ CD4- CD8+). B lymphocytes were identified as CD3- CD20+ cells.

### 2.9 Cytokine quantification

Seminal plasma concentrations of IL-8, TNF, IL-1β, IL-6, IFNγ, IL-10, and IL-17A were analyzed by enzyme-linked immunosorbent assay (ELISA) using specific kits and according to the manufacturers’ instructions. IL8-, TNF, IL-6, IFNγ, and IL-10 were respectively quantitated by BD OptEIA specific ELISA sets (BD Biosciences Pharmingen, San Diego, CA, USA). IL-17A and IL-1β were respectively assayed by ELISA Ready-SET-Go specific kits (eBioscience, San Diego, CA, USA). Samples were analyzed at least in triplicates, with results expressed as pg/ml. In the case of IL-8 quantitation, seminal plasma samples were assayed diluted 1/20.

### 2.10 Statistical analysis

Statistical analysis was performed using the GraphPad Prism software, version 9.0 (GraphPad Inc., La Jolla, CA, USA) and the SPSS Statistics for Windows software, version 25.0 (IBM, Armonk, NY, USA). Data distribution was assessed by the Shapiro-Wilk test. Data were analyzed using the Mann-Whitney test or Kruskal-Wallis test with the Dunn’s multiple comparisons *post hoc*. Differences were considered statistically significant when *p*<0.05. Compromise power analyses was calculated using G*Power3 data analysis software. Considering ρ: 0.19 and α: 0.05, the statistical power of the study (1-β) was 0.867 (86.7%).

## 3 Results

### 3.1 Characterization of genital infection by HPV and other common uropathogens in men attending a urology clinic

**Figure 2:**
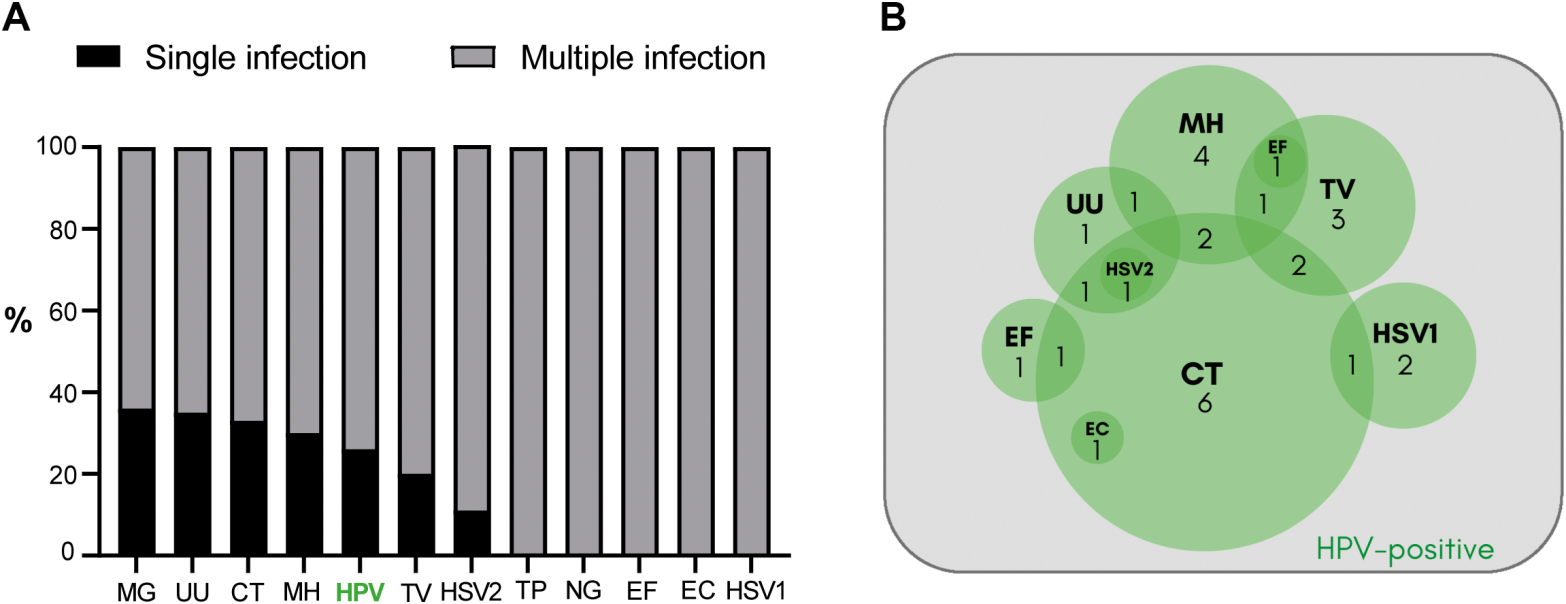
Characterization of the urogenital infection by HPV and other common uropathogens in a cohort of sexually adult young adult men attending a urology clinic. **(A)** The bar graph illustrates the coinfection rates detected according to the uropathogens analyzed. The percentage of single infection is depicted in black and the percentage of multiple infection with ≥2 pathogens detected is shown in gray. MG, *Mycoplasma genitalium*; UU, *Ureaplasma urealyticum*; CT, *Chlamydia trachomatis*; MH, *Mycoplasma hominis*; HPV, Human Papillomavirus; TV, *Trichomonas vaginalis*; HSV2, Herpes simplex virus type 2; TP, *Treponema pallidum*; NG, *Neisseria gonorrhoeae*; EF, *Enterococcus faecalis*; EC, *Escherichia coli*; HSV1, Herpes simplex virus type 1. **(B)** The Venn diagram illustrates the cases of HPV-associated coinfections. Coinfection cases with one other uropathogen: 6 HPV-CT, 4 HPV-MH, 3 HPV-TV, 2 HPV-HSV1, 1 HPV-EF, and 1 HPV-UU. Coinfection cases with 2 other uropathogens: 2 HPV-CT-TV, 2 HPV-CT-MH, 1 HPV-CT-HSV1, 1 HPV-CT-UU, 1 HPV-CT-EF, 1 HPV-CT-EC, 1 HPV-MH-TV, 1 HPV-MH-UU. Coinfection cases with more than 2 other uropathogens: 1 HPV-MH-EF-TV and 1 HPV-CT-UU-HSV2.

The median age of the 205 male participants under study was 35 years (95% CI: 34–36 years old). An exhaustive analysis of HPV infection among several other common uropathogens in semen was performed. Strikingly, the analysis showed that 76.1% of patients (n=156) were positive for infection with at least one of the uropathogens analyzed. Subsequently, it was determined how often these uropathogens were present as single infection or as multiple infection with other/s uropathogens/s. As shown in **Figure 2A**, infections were detected as coinfections with one or more other uropathogens in most cases. The overall rate of coinfection ranged 64-100%. Noteworthy, pathogens such as HPV, MG, UU, CT, TV and HSV2 were detected either as single infections or coinfections with other uropathogens, whereas TP, NG, EF, EC and HSV1 were detected coinfecting with other uropathogens in all cases analyzed (**Figure 2B**). The overall prevalence of HPV infection was 19.0% (39 out of 205 patients), with 25.6% of cases as single infections (10/39) and the remaining 74.4% of cases as coinfection with one or more of the other uropathogens tested (29/39) (**Figure 2A**). **Figure 2B** shows the complexity of HPV-associated coinfections. Among the 29 cases in which HPV was detected coinfecting with other pathogens, in 17 cases HPV was coinfecting with only one additional pathogen (HPV-CT: 6 cases, HPV-MH: 4 cases, HPV-TV: 3 cases, HPV-HSV1: 2 cases, HPV-EF: 1 case, and HPV-UU: 1 case). In 10 cases, HPV was found coinfecting with 2 other pathogens (HPV-CT-TV: 2 cases, HPV-CT-MH: 2 cases, HPV-CT-HSV1: 1 case, HPV-CT-UU: 1 case, HPV-CT-EF: 1 case, HPV-CT-EC: 1 case, HPV-MH-TV: 1 case, HPV-MH-UU: 1 case). In the remaining 2 HPV+ cases, 3 pathogens along with HPV were found (HPV-MH-EF-TV and HPV-CT-UU-HSV2). Although HPV male urogenital infection showed a high rate of coinfection, regression analysis showed that it was not particularly associated with multiple coinfections; i.e., the presence of 2 or more coinfections in parallel (*p*= 0.704, OR:0.85; 95% CI: 0.37-1.94).

These findings indicate that HPV infection is highly prevalent among young adult men attending a urology clinic. Interestingly, it often occurs as coinfection with other common uropathogens. Particularly, the coinfection of HPV with CT is a distinctive feature, as CT was present in 15 out of 29 (51.7%) cases of HPV coinfection.

### 3.2 Analysis of sperm quality, semen inflammation and oxidative stress in men with HPV urogenital infection

Subsequently, it was analyzed whether HPV infection, either as single infection or coinfection with other uropathogens, was associated with alterations in sperm quality. For this purpose, patients with HPV infection were classified into two groups: one encompassing those individuals positive for HPV infection only (HPV+, n=10) and another including patients infected with HPV plus one or more other uropathogens tested (HPV+Coinf+, n=29). Individuals negative for all analyzed uropathogens, and without leukocytospermia, were selected as controls (control individuals, n=43).

No significant differences in routine sperm quality parameters, such as ejaculate volume, sperm concentration, total and progressive sperm motility, sperm morphology, and sperm viability (assessed by eosin staining), were observed between patients with HPV infection alone, those with HPV and other uropathogens (HPV+Coinf+), and control individuals (**Table 1**). When analyzing sperm apoptosis/necrosis, no significant differences were found in the frequencies of live sperm between controls and both groups of HPV infected individuals. Interestingly, significantly lower levels of early (AnV+PI-) and late (AnV+PI+) apoptotic sperm were observed in HPV+Coinf+ group compared to controls (**Table 1**). Furthermore, HPV+ individuals showed significantly lower levels of early apoptotic (AnV+PI-) sperm and higher levels of necrotic (AnV-PI+) sperm with respect to controls (**Table 1**).

**Table 1:** Sperm quality in male patients bearing HPV urogenital infection with or without coinfections.

Sperm quality	Control individuals (n=43)	HPV+ patients (n=10)	*p- value ^a^*	HPV+ Coinf+ patients (n=29)	*p- value ^b^*
Sperm parameters					
Volume (ml/ejaculate)	2.8 ± 1.4	2.8 ± 0.5	0.514	2.8 ± 1.4	0.993
Sperm concentration (x10^6^/ml)	102.7 ± 78.7	87.9 ± 72.7	0.731	114.7 ± 99.7	0.813
Eosine viability (%)	87.6 ± 5.6	84.7 ± 6.8	0.232	85.6 ± 7.1	0.202
Total motility (PR+NP, %)	53.3 ± 14.4	42.8 ± 15.5	0.085	44.3 ± 18.9	0.098
Progressive motility (PR, %)	52.9 ± 14.4	42.8 ± 15.5	0.098	43.8 ± 19.1	0.093
Normal morphology (%)	6.1 ± 3.1	4.2 ± 2.7	0.077	4.8 ± 2.5	0.089
Sperm viability (AnV/PI)					
Live sperm (AnV-/IP-)	57.6 ± 16.4	59.0 ± 19.6	0.656	62.7 ± 13.9	0.321
Early apoptotic sperm (AnV+/IP-)	9.5 ± 5.7	4.1 ± 3.4	**0.003**	6.7 ± 5.9	**0.014**
Late apoptotic sperm (AnV+/IP+)	18.9 ± 9.3	12.8 ± 6.8	0.089	13.4 ± 8.0	**0.028**
Necrotic Sperm (AnV-/IP+)	11.0 ± 6.9	21.1 ± 16.0	**0.048**	14.0 ± 8.9	0.095

HPV+ patients: all patients positive for HPV and negative for all other uropathogens analyzed; HPV+Coinf+ patients: HPV positive patients with a co-infection by at least one of the uropathogens analyzed; Control individuals: Individuals without leukocytospermia and negative for all analyzed uropathogens. Total motility is the sum of progressive motility (PR) and non-progressive motility (NP, data not shown). *p*-values were calculated using the Mann-Whitney test. Comparisons were performed between HPV+Coinf- group (^a^) or HPV+Coinf+ patients (^b^) and the control group. Data are shown as Mean ± SD. Bold numbers indicate statistically significant differences when *p* < 0.05

Next, the putative association of HPV single infection or coinfection with other uropathogens with semen inflammation and oxidative stress was evaluated. Sperm ROS production and semen levels of peroxidase-positive cells, inflammatory cytokines and counts of leukocyte subsets were assessed. Interestingly, significantly higher frequencies of ROS+ spermatozoa were observed in HPV+Coinf+ individuals (73.0 ± 20.0) when compared with controls (50.0 ± 29.0; *p*=0.010, **Table 2**). However, no significant differences in the frequencies of ROS+ spermatozoa were found between HPV single infected patients (82.2 ± 29.1) and controls (50.0 ± 29.0; *p*=0.063, **Table 2**). In addition, we found no differences in semen counts of peroxidase-positive cells between patients only infected with HPV (HPV+) or coinfected with other uropathogens (HPV+Coinf+) and control individuals (**Table 2**). Since this cytochemical assay detects neutrophils and activated macrophages without considering other leukocyte subsets, the counts of total leukocytes were assessed by CD45 staining (a panleukocytic biomarker that identifies all leukocyte subsets) and flow cytometry. Strikingly, HPV+ patients displayed significantly lower semen CD45+ cell counts than control individuals, whereas no such difference was observed between HPV+Coinf+ patients and the control group (**Table 2**). However, when analyzing in further detail different leukocyte subpopulations in semen, no significant differences in the levels of any particular cell subset were observed between HPV+ patients and controls (**Table 2**). These data indicate that the decrease in the overall count of semen leukocytes in patients infected only with HPV was not due to a decrease in a specific subpopulation type, such as neutrophils, lymphocytes, or monocytes. Instead, it was likely due to an overall reduction in the number of all subtypes of leukocytes (**Table 2**).

**Table 2:** Local inflammation biomarkers in semen from male patients bearing HPV urogenital infection with or without coinfections.

Inflammation biomarkers	Control individuals (n=43)	HPV+ patients (n=10)	*p- value ^a^*	HPV+ Coinf+ patients (n=29)	*p- value ^b^*
ROS+ spermatozoa (%)	50.0 ± 29.0	82.2 ± 29.1	0.063	73.0 ± 20.0	**0.010**
Peroxidase-positive cells (x10^5^/ml)	2.1 ± 2.9	0.8 ± 0.8	0.367	2.5 ± 4.3	0.960
CD45-positive cells (x10^5^/ml)	6.75 ± 10.3	1.3 ± 1.5	**0.039**	6.9 ± 10.4	0.838
PMN leukocytes (x10^3^/ml)	148.8 ± 214.6	70.9 ± 65.3	0.672	157.5 ± 287.3	0.578
Monocytes (x10^3^/ml)	64.1 ± 114.4	26.7 ± 24.4	0.910	83.8 ± 137.4	0.981
Lymphocytes (x10^3^/ml)	47.9 ± 98.9	6.0 ± 8.3	0.309	18.8 ± 26.7	0.810
T cells (x10^3^/ml)	16.9 ± 46.7	1.1 ± 1.9	0.103	11.7 ± 22.8	0.966
CD4 T cells (x10^3^/ml)	3.6 ± 9.8	0.1 ± 0.2	0.131	6.1 ± 15.9	0.997
CD8 T cells (x10^3^/ml)	3.0 ± 4.9	0.3 ± 0.6	0.121	2.3 ± 4.3	0.356
CD4/CD8 T cells ratio	1.4 ± 1.9	2.05 ± 2.1	0.431	6.4 ± 18.6	0.367
B cells (x10^3^/ml)	2.2 ± 4.6	0.3 ± 0.4	0.754	0.3 ± 0.4	0.582
Cytokines in seminal plasma (pg/ml)					
IL-8	3379 ± 2193	3456 ± 2171	0.411	3312 ± 3450	0.163
IL-6	126.6 ± 137.4	12.17 ± 5.78	**0.018**	89.01 ± 103.1	0.329
IL-1β	8.3 ± 7.41	7.56 ± 7.44	0.571	7.05 ± 8.47	0.110
TNF	11.53 ± 10.85	10.96 ± 5.81	0.419	11.53 ± 9.15	0.420
INF-γ	24.03 ± 19.01	27.24 ± 30.31	0.881	29.44 ± 16.19	0.235
IL-10	9.55 ± 9.14	8.94 ± 8.67	0.653	10.79 ± 12.41	0.982
IL-17	13.55 ± 12.94	10.47 ± 9.67	0.634	15.26 ± 11.68	0.357

HPV+ patients: all patients positive for HPV and negative for all other uropathogens analyzed; HPV+Coinf+ patients: HPV positive patients with a co-infection by at least one of the uropathogens analyzed; Control individuals: Individuals without leukocytospermia and negative for all analyzed uropathogens. Leukocyte population analyses were conducted using flow cytometry within gates defined on the total leukocyte cell population (CD45+). The subpopulations identified included polymorphonuclear leukocytes (PMN, CD11c-CD11b+), monocytes (CD14+CD11b-), and lymphocytes (CD11c-, CD11b-). T cells were characterized as CD3+ lymphocytes, with further subtyping into CD4 T cells (CD3+ CD4+ CD8-) and CD8 T cells (CD3+CD4-CD8+), along with B cells (CD3-CD20+). The results were reported as absolute leukocyte counts. *p*-values were calculated using the Mann-Whitney test. Comparisons were performed between HPV+Coinf- group (^a^) or HPV+Coinf+ patients (^b^) and the control group. Data are shown as Mean ± SD. Bold numbers indicate statistically significant differences when *p* < 0.05.

Semen inflammation was further assessed by quantifying levels of different cytokines in seminal plasma. Comparable levels of most of the cytokines analyzed (IL-1β, IL-8, TNF, IFN-γ, IL-17A and IL-10) were observed between HPV+, HPV+Coinf+ individuals and controls (**Table 2**). Interestingly, HPV+ patients showed markedly reduced semen levels of the inflammatory cytokine IL-6 with respect to controls; however, this effect was not observed in HPV+Coinf+ individuals (**Table 2**). These findings suggest that, rather than triggering inflammation, HPV infection itself induces an anti-inflammatory environment characterized by a decrease in local leukocyte recruitment and reduced levels of IL-6.

### 3.3 Analysis of sperm quality and biomarkers of local inflammation during HPV-CT coinfection

**Figure 3:**
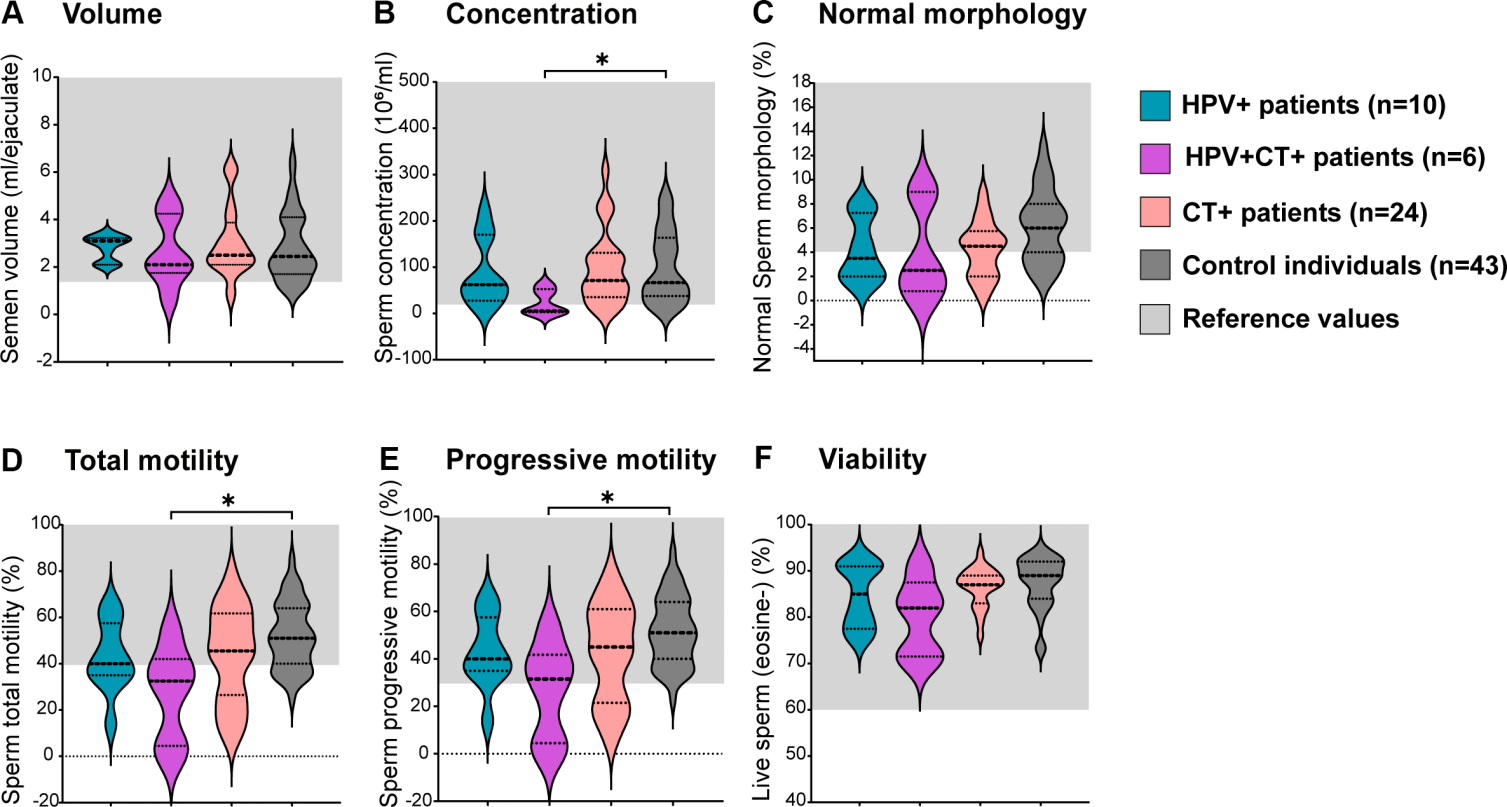
Analysis of sperm quality in male patients bearing HPV or HPV-Chlamydia trachomatis urogenital infections and in non-infected control individuals.Violin plots display classic sperm quality parameters analyzed: sperm volume **(A)**, concentration **(B)**, normal morphology **(C)**, total motility **(D)**, progressive motility **(E)** and viability **(F)**. Dotted lines indicate median and interquartile ranges. Reference ranges following the WHO 2010 criteria are shown in light gray shading. Comparisons were performed between the following groups: control individuals (dark gray) negative for all analyzed uropathogens without leukocytospermia, HPV+ patients (turquoise): HPV positive patients without any other screened infection, HPV+CT+ patients (purple): Patients positive for HPV and *Chlamydia trachomatis* co-infection but negative for any other uropathogens screened; CT+ patients (pink): *Chlamydia trachomatis* positive patients without any other screened infection. Comparisons were performed between the four groups, *p*-values were calculated using the Kruskal–Wallis non-parametric test with the Dunn’s multiple comparisons *post hoc*. Differences were considered statistically significant when *p* < 0.05. The * means the statistical significance (*p* < 0.05).

Given that CT was the most frequently detected uropathogen coinfecting with HPV, a comparative analysis was conducted among individuals with HPV infection only (HPV+), CT infection only (CT+), HPV and CT coinfection (HPV+CT+) and controls. Interestingly, no significant differences were observed in ejaculate volume, sperm morphology, total or progressive motility and viability between HPV+ or CT+ and controls (**Figure 3**). However, patients bearing the coinfection with HPV and CT (HPV+CT+) showed significantly decreased sperm concentration, as well as reduced total and progressive sperm motility, compared to controls (**Figure 3**). Nevertheless, no significant differences in the levels of sperm apoptosis/necrosis were observed among groups (**Supplementary Figure S2**).

**Figure 4:**
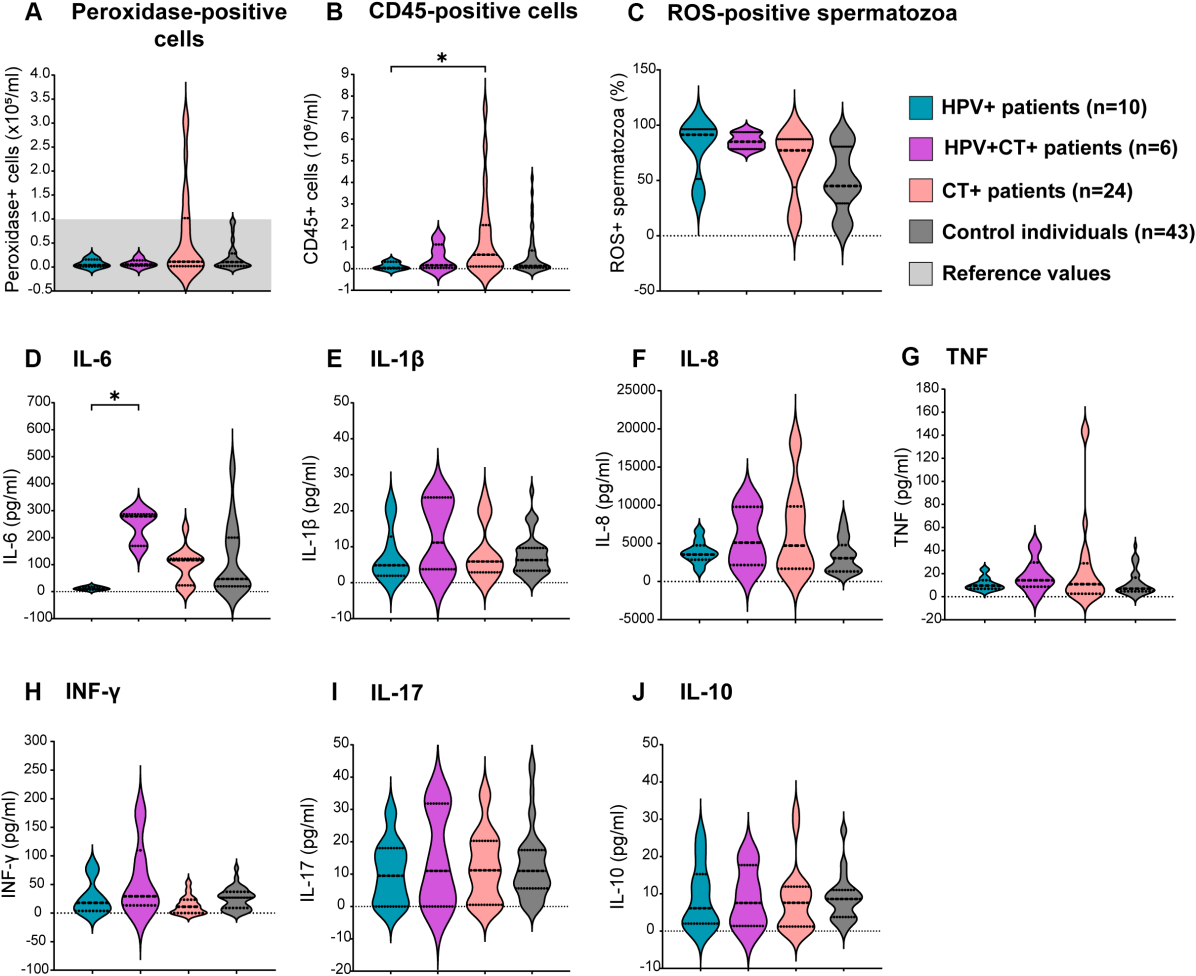
Assessment of sperm oxidative stress and semen inflammation in patients bearing HPV or HPV-Chlamydia trachomatis urogenital infections and non-infected control individuals. Violin plots display results from the assessment of different biomarkers of semen inflammation and oxidative stress in patients under study. Dotted lines in the graph represent medians and interquartile ranges. Total leukocytes were analyzed using **(A)** peroxidase cytochemical staining or **(B)** the quantitation of cells expressing the pan-leukocyte marker CD45 (total leukocytes) in semen by flow cytometry. **(C)** Frequencies of Reactive Oxygen Species (ROS)-positive spermatozoa evaluated by flow cytometry using the Dcfh-DA probe. **(D–J)** Levels of different cytokines in seminal plasma analyzed at least in triplicates by sandwich ELISA. Comparisons were performed between the following groups: control individuals (dark gray) negative for all analyzed uropathogens without leukocytospermia, HPV+ patients (turquoise): HPV positive patients without any other screened infection, HPV+CT+ patients (purple): Patients positive for HPV and *Chlamydia trachomatis* coinfection but negative for any other uropathogens screened, CT+ patients (pink): *Chlamydia trachomatis* positive patients without any other screened infection. *p*-values were calculated using the Kruskal–Wallis non-parametric test with the Dunn’s multiple comparisons *post hoc*. Differences were considered statistically significant when *p* < 0.05. The * means the statistical significance (*p* < 0.05).

To elucidate whether the single infections by HPV or CT, or the HPV-CT coinfection, were associated with semen inflammation and oxidative stress, levels of sperm ROS production, semen cytokines and leukocyte counts were assessed in the semen samples. Significantly elevated counts of total leukocytes (CD45+ cells) were observed in the semen of patients bearing CT single infection (CT+ patients) with respect to those infected only with HPV (HPV+ patients, **Figure 4B**). Although non-significant, a similar trend was noted for peroxidase-positive cell counts in semen (**Figure 4A**). However, comparable levels of either total leukocytes (CD45+ cells) or peroxidase-positive cells were observed among patients bearing HPV-CT coinfection (HPV+CT+ patients), HPV single infection (HPV+ patients) and control individuals (**Figures 4A, B**). When analyzing the leukocyte subpopulations, no significant differences between the groups were found (data not shown). Additionally, comparable levels of sperm ROS production were observed among all groups (**Figure 4C**). Furthermore, no significant differences were found in the seminal levels of most of the cytokines analyzed (IL-1β, IL-8, TNF, IFNγ, IL-17 and IL-10), among the groups (**Figures 4E–J**). Nevertheless, significantly higher seminal levels of IL-6 were detected in patients coinfected with HPV and CT (HPV+CT+) with respect to those bearing a single infection with HPV (HPV+, **Figure 4D**).

These results indicate that the HPV infection alone does not associate with significant alterations in sperm quality. However, the coinfection with CT is associated with increased semen levels of IL-6 and significantly decreased sperm concentration and progressive and total motility.

## 4 Discussion

The study of coinfections is gaining increasing interest in current clinical practice, as screening for a single particular infection and analyzing its association with different biomarkers is a limited approach. Despite the rising frequencies of coinfections, their implications in disease development and their consequences remain to be fully elucidated (Dalby and Stoner, 2022; Lee et al., 2023). A recent study involving a cohort of 65,191 men and women found that 54.3% of participants had one or more STIs, with notably high rates of coinfections (Lee et al., 2023). Particularly, HPV infection and coinfection with other uropathogens has been more extensively investigated in women, in whom coinfections appear to play a key role in cervical cancer progression and HPV persistence (Alotaibi et al., 2020; Cunha et al., 2020; Ciccarese et al., 2021). In the case of HPV related coinfections of the male genital tract, the scarce reported data mainly come from investigations focused on the potential oncogenic risk of these associated infections in particular patient populations, including HIV-positive males or men who have sex with men (MSM) (Li et al., 2016; Comar et al., 2017; Masiá et al., 2020). However, Gimenes et al. reported a markedly elevated prevalence of HPV genital infection and high rates of coinfections in male partners of infertile couples (Gimenes et al., 2014b). In agreement, results from the present study revealed a high prevalence of urogenital infections (76.0%) in young adult men seeking care for couple’s infertility or LUTS. Moreover, in accordance with Gimenes et al (Gimenes et al., 2014b), a high prevalence of HPV male urogenital infection (19.0%) and elevated rates of coinfection with other uropathogens were found. Interestingly, in the present study, HPV was identified coinfecting with a single other uropathogen instead of multiple coinfections in most cases. Noteworthy, this study provides a comprehensive analysis of HPV related coinfections in a population of sexually active men who are not classified as high-risk of acquiring STIs.

The high prevalence of HPV related coinfections observed is a critical aspect to consider since HPV urogenital infection has been proposed to impair male fertility. The concurrent infection with different uropathogens can lead to heightened infectivity, exacerbated symptoms, increased likelihood of pathogen transmission and even masked effects produced by one or another pathogen (Gimenes et al., 2014b). The possibility to identify a single HPV infection in patients, excluding the coinfection with other common uropathogens, allows concluding that HPV infection of the male genital tract would not significantly affect routinely assessed sperm quality parameters, semen inflammation or oxidative stress. However, more research on this topic is needed to corroborate our findings. The results presented contrast with studies that have reported decreased sperm concentration and motility in men bearing HPV urogenital infection (Boeri et al., 2019; Moghimi et al., 2019). However, those studies did not thoroughly evaluate other possible coinfections, which could have accounted for the observed alterations more than HPV infection itself. Besides, those dissimilarities could be due to variations in the study design, patient populations analyzed and/or detection methodologies.

Interestingly, although the present study revealed no alterations in conventional sperm parameters in patients with single HPV infection, higher levels of necrotic spermatozoa and a tendency toward higher sperm ROS production were observed in these patients. Notably, significantly higher levels of sperm ROS production were found in patients coinfected with HPV plus other uropathogens with respect to control individuals. In that regard, it has been reported that HPV induces oxidative stress and DNA damage in epithelial cells (De Marco, 2013; Williams et al., 2014). Moreover, Perez-Soto et al. reported that men bearing urogenital infection with HPV showed significantly increased levels of sperm lipid peroxidation and decreased expression of the antioxidant enzymes catalase and superoxide dismutase. Besides, these effects were more pronounced in those individuals bearing infections with multiple high-risk HPV genotypes (Pérez-Soto et al., 2022). However, these authors only screened for infections with different HPV genotypes and overlooked the impact of potential concurrent infections with other uropathogens. These findings underscore the need to further investigate whether HPV is per se able to trigger sperm oxidative stress and the involved mechanisms, given that oxidative stress and decreased sperm DNA integrity have well-known detrimental consequences on male fertility (Henkel et al., 2021).

On the other hand, no semen inflammation was detected in patients bearing HPV infection alone. On the contrary, and unexpectedly, markedly reduced semen counts of CD45+ leukocytes and IL-6 levels were observed in these patients. These results indicate that HPV male urogenital infection is related to a biased immune response toward no semen inflammation. Supporting that, it has been reported that HPV employs multiple mechanisms to evade the immune system (Bordignon et al., 2017; Barros et al., 2018; Zhou et al., 2019). These mechanisms collectively result in reduced recruitment of immune cells to infected tissues, delaying the initiation of anti-HPV immune responses, and potentially facilitating coinfections with other sexually transmitted pathogens and HPV persistence.

Remarkably, data presented herein revealed that CT was the most frequently detected uropathogen coinfecting with HPV. Consistent with this, several studies have found an association between CT infection or history of CT infection with increased risk of HPV infection, both in men and women (Bellaminutti et al., 2014; Comar et al., 2017; Olivera et al., 2021). In fact, it has been previously reported that men with urogenital CT infection are almost 12 times more likely to have HPV infection than CT-negative individuals (Olivera et al., 2021). In addition, Shigehara et al. indicated that CT infection is an independent risk factor for HPV infection in men with urethritis (Shigehara et al., 2011). The partnership between HPV and CT may be explained by shared features such as common transmission route, commonly asymptomatic nature favoring transmission, and same infection risk factors (Gimenes et al., 2014a; Stojanov et al., 2018). Moreover, both pathogens are characterized by developing chronic persistent infections, and exerting mechanisms of immune evasion that benefit themselves at expense of the host (Li et al., 2009; Niebler et al., 2013; Ainouze et al., 2018; Chiang et al., 2018; Karim et al., 2018; Panzetta et al., 2019; Sanchez et al., 2019; Wong et al., 2019; Brockett and Liechti, 2021; Castagnino et al., 2022). Some studies have reported that CT urogenital infection in men is associated with decreased sperm concentration, motility, and morphology, as well as increased levels of certain inflammatory biomarkers (Sellami et al., 2014; Dehghan Marvast et al., 2016; Ahmadi et al., 2023). However, other authors reported no association of CT male urogenital infection with sperm quality impairment (López-Hurtado et al., 2018; Farahani et al., 2021). The present study revealed that patients bearing infection with CT alone have no significant alterations in semen quality. Still, patients bearing urogenital coinfection with HPV and CT showed alterations in sperm quality such as significantly decreased sperm concentration and progressive and total motility. These results support previously reported data by *Cai* et al., indicating that HPV-CT coinfection is associated with significantly impaired male fertility potential in heterosexual men experiencing chronic prostatitis symptoms (Cai et al., 2014).

Although some studies have reported a relationship between male urogenital CT infection and semen inflammation (Al-Sweih et al., 2012; López-Hurtado et al., 2018), others have found contrary results (Yoshida et al., 1994; Kokab et al., 2010). Data presented herein revealed significantly higher counts of leukocytes in the semen from patients with CT infection alone compared to those bearing only HPV infection. However, no significant differences were found between HPV+ or CT+ patients and control individuals. In addition, increased levels of IL-6 were detected in semen from HPV-CT coinfected patients with respect to patients only infected with HPV. Conversely, while HPV infection alone associated with reduced seminal IL-6 levels, CT single infection associated with increased seminal IL-6 levels. These findings suggest that in the male urogenital coinfection with HPV and CT, the combined effect of both pathogens promotes the local secretion of IL-6. Although we cannot explain this dissimilar association with the sole HPV infection with respect to the HPV-CT infection, our results suggest a putative synergistic effect between the two uropathogens in triggering IL-6 secretion. Interestingly, reported data indicates that CT infection associates with increased levels of IL-1β and IL-6 in semen, thus suggesting a potential role of infection on promoting inflammation in asymptomatic patients (Pérez-Soto et al., 2021b). Moreover, these data indicate that CT, unlike HPV, may in some cases promote leukocyte recruitment during coinfection. The biological mechanisms underlying the particular consequences that the HPV-CT coinfection would have on semen inflammation, oxidative stress and sperm quality, with respect to CT or HPV single infections, remain unexplored. In fact, to our knowledge, there are scarce to absent reported data on the HPV-CT coinfection of the male genital tract, thus revealing a nascent research field that needs to be explored in detail to provide a better understanding of this unmet clinical need.

Our study has some limitations. First, the observed associations do not mean causality, which remain to be established by further research employing different experimental strategies and models. Second, while patients and controls studied were carefully enrolled according to several inclusion and exclusion criteria, potential confounding factors not assessed, including previous sexually transmitted infections, unknown toxic exposures, or the concomitant presence of other rare or uncommon infections, could have biased our results. Third, knowing the infecting HPV genotypes would have provided valuable additional information to allow a better understanding of the putative implications of HPV sole infection or its coinfections with the other uropathogens analyzed and their association with sperm quality, oxidative stress, and inflammation.

In conclusion, the present study revealed a considerable prevalence of HPV infection and a high rate of coinfection with other known uropathogens, mainly CT, in sexually active adult men attending a urologic clinic. Moreover, single HPV and CT infections, and HPV/CT coinfection, are differentially associated with semen inflammation and sperm quality. Although definite causal relationships remain to be established, the HPV-CT coinfection appears to be a clinical entity with its own features different from those of individual HPV or CT infections, in which each pathogen would seek to conveniently and differentially modulate cellular metabolism and the immune response. These findings have important implications for the screening and care of patients with male urogenital infections. First, the high rate of HPV coinfection with other different uropathogens detected with respect to HPV single infections indicate clinicians should consider screening several other uropathogens besides HPV only. Moreover, special attention should be paid to the screening of the concurrent HPV and CT infection since it could represent a valuable intervention to prevent severe reproductive health complications and sequelae such as cervical cancer and infertility. Nevertheless, our results should be interpreted with caution. Additional studies including larger patient populations are needed to further support our data. In addition, longitudinal studies assessing infection/coinfections persistence and potential long-term reproductive consequences will allow a better understanding of these prevalent clinical conditions.

## Data Availability

The original contributions presented in the study are included in the article/**Supplementary Material**. Further inquiries can be directed to the corresponding authors.
