# Supplementary material for: HPV and co-infections: impacts on semen inflammation, oxidative stress, and sperm quality
Source: Front Cell Infect Microbiol. 2025 Mar 26;15:1539871. doi: 10.3389/fcimb.2025.1539871 (PMC11979249; doi:10.3389/fcimb.2025.1539871)
Supplement: Supplementary file 1 [file DataSheet1.pdf]

Supplementary Material

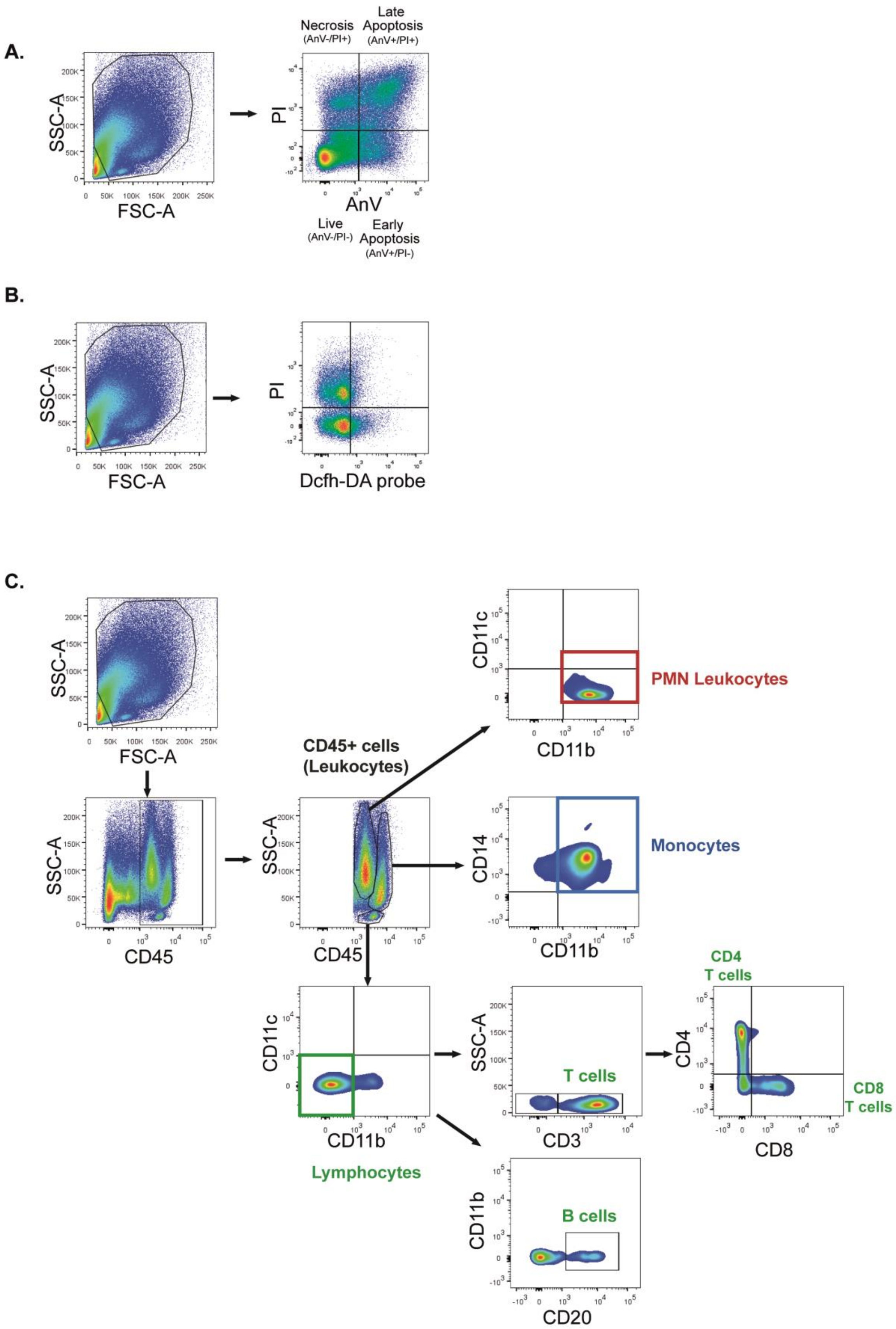
A.
SSC-A
FSC-A
PI
AnV
Necrosis
(AnV-/PI+)
Late
Apoptosis
(AnV+/PI+)
Live
(AnV-/PI-)
Early
Apoptosis
(AnV+/PI-)
B.
SSC-A
FSC-A
PI
Dcfh-DA probe
C.
SSC-A
FSC-A
CD45
CD45+ cells
(Leukocytes)
CD11c
CD11b
PMN Leukocytes
CD14
Monocytes
Lymphocytes
CD3
T cells
CD4
T cells
CD8
T cells
CD4
CD8
B cells
CD20

Supplementary Figure 1. *Gating strategies used for the assessment of sperm apoptosis/necrosis, sperm oxidative stress, and leukocyte subpopulation in semen by flow cytometry*. (A) Representative dot plots showing the gating strategy used for the assessment of sperm apoptosis/necrosis by Annexin V (AnV) and Propidium iodide (PI) staining, showing live sperm (AnV-PI-), early apoptotic sperm (AnV+PI-), late apoptotic sperm (AnV+PI+), and necrotic sperm (AnV-PI+). (B) Representative dot plots showing the gating strategy used for the assessment of sperm ROS production by the staining with the cell-permeable probe 2’,7’-dichlorodihydrofluorescein diacetate (Dcfh-DA) and PI as a supravital stain. Live and dead ROS-positive spermatozoa are shown as Dcfh+PI- and Dcfh+PI+, respectively. (C) Representative dot plots showing the gating strategy used for the assessment of total leukocytes (CD45+) and leukocyte subpopulations in semen. The analyses were performed on total leukocytes (CD45+) using light side scatter (SSC) versus CD45 plots, where the subpopulations of lymphocytes (CD11c-CD11b-), monocytes (CD14+CD11b-), and polymophonuclear cells (PMN) (CD11c-CD11b+) were selected. From lymphocytes (CD11c-CD11b-), subpopulations of T cells (CD3+), B cells (CD3-CD20+), T helper cells (CD3+CD4+CD8-), T cytotoxic cells (CD3+CD4-CD8+), and NKT and other cells (CD3+CD4-CD8-) were selected. Total cells/events in 100 microliters of sample were analyzed. The results were expressed as % of cells.

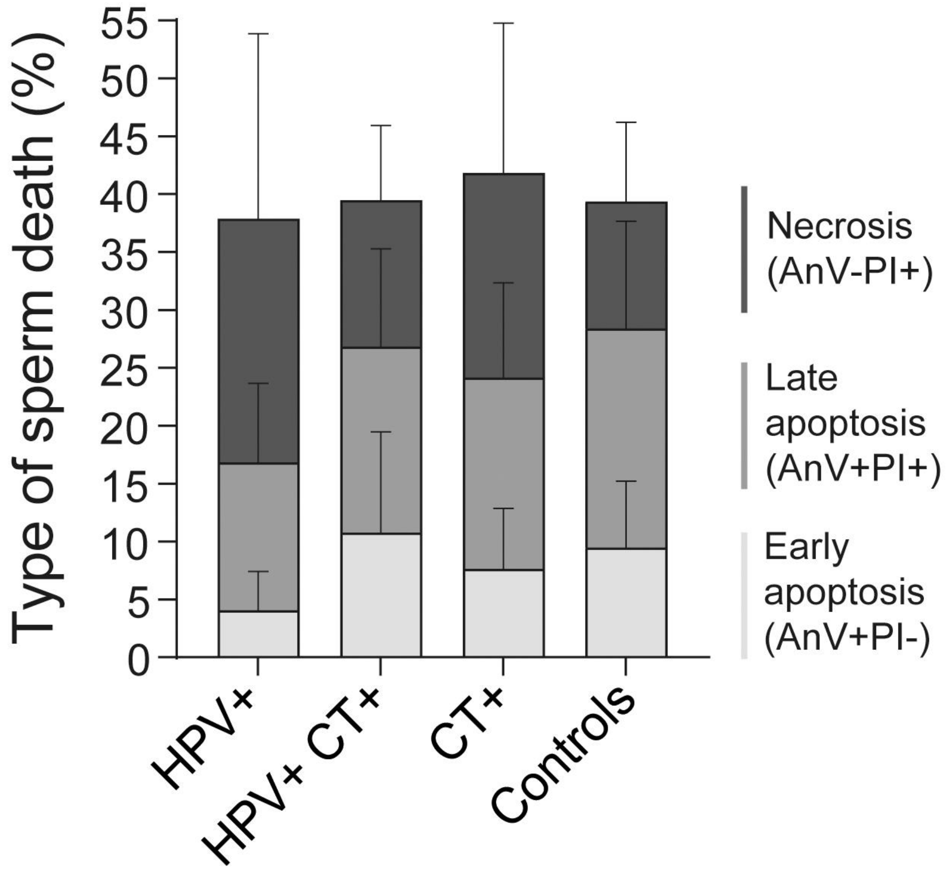

Supplementary Figure 2. *Assessment of sperm death mechanisms in patients bearing HPV and/or Chlamydia trachomatis urogenital infections and non-infected control individuals.* Sperm apoptosis/necrosis was analyzed using Annexin (AnV)/Propidium Iodide (PI) staining by flow cytometry. Comparisons were perfomed between the following groups: HPV+ patients (n=10, HPV positive patients without any other screened infection), HPV+CT+ patients (n=6, patients positive for HPV and *Chlamydia trachomatis* coinfection but negative for any other uropathogens screened), CT+ patients (n=24, *Chlamydia trachomatis* positive patients without any other screened infection), and control individuals (n=43, individuals negative for all analyzed uropathogens without leukocytospermia). Data are shown as Mean ± SD. *p*-values were calculated using the Kruskal-Wallis non-parametric test. Differences were considered statistically significant when $p < 0.05$.
